# Supplementary material for: A macroscopic link between interhemispheric tract myelination and cortico-cortical interactions during action reprogramming
Source: Nat Commun. 2022 Jul 22;13:4253. doi: 10.1038/s41467-022-31687-5 (PMC9307658; doi:10.1038/s41467-022-31687-5)
Supplement: Supplementary file 1 — Supplementary Information [file 41467_2022_31687_MOESM1_ESM.pdf]

Supplementary Methods

Transcranial Magnetic Stimulation (TMS) set-up

Two DuoMAG MP-Dual TMS monophasic stimulators (DeyMed DuoMag, Rogue Resolutions Ltd.) were used to deliver paired pulses via two figure-eight coils, one 70mm-diameter coil over left M1 and one 50mm-diameter coil over right PMv.

The 70mm/M1 coil was held by the main experimenter throughout the experiment. This coil was kept tangential to the skull and at roughly an 45° angle to the scalp's midline, resulting in a Posterior-to-Anterior (PA) current direction induced in the cortex. The 50 mm PMv coil was positioned in place through a clamp sustained by a Manfrotto Variable Friction Arm (Wex Photo Video, Calumet Photographic Limited) which was clamped to the experiment table. The position of the coil was determined by registration of its location to the participant's T1w image, and targeted coordinates x = 58, y = 15, z = 30 in MNI (Montreal Neurological Institute) space, based on previous literature[1]. The angle of the PMv coil was 0° relative to midline.

Participants sat on a chair and were asked to position their head on a chin rest in order to minimize head movement. Before the start of any TMS stimulation, participants were asked to keep their feet relaxed and flat on the ground. The participants were free to move their body between task blocks, but they were asked to move the hand with the electrodes as little as possible both during the task and between task blocks. All participants wore earplugs to reduce the effects of TMS-related noise.

Using Neuronavigation to track stimulation sites

All stimulation was delivered using continuous tracking of coil location with respect to subject neuroanatomy (i.e. neuronavigation). This was achieved through a Polaris camera and the Brainsight (Rogue Resolutions, Inc.) software. The participant was tracked via a headband with reflective spheres

attached to it; the coils were tracked with coil trackers that were re-calibrated at the beginning of each testing day. In addition, extensive hard-ware checks were performed before each session, including that the coils worked on a peripheral muscle, that cable connections were correctly in place, and importantly, a PicoScope6 (Pico Technology) was used to check the timing of the two pulses to ensure they were correct and identical between task and rest blocks at sub-millisecond precision.

Online neuronavigation was used in all subjects to ensure the coil was targeting the cortical area of choice throughout the task. Moreover, a sample of the coil location was collected for each participant during the session, and analysed offline. An automated Brainsight tool was used to find the closest brain voxel to the sampled stimulation site. The coordinates for this voxel were then transformed in standard space to allow overlaying of stimulation sites from different participants. At this stage, a total of 47 stimulation locations were included, as two participants' stimulation locations failed to save due to software fault, three participants's stimulation locations were not sampled due to time limits, and four participant's stimulation locations could not be automatically determined with Brainsight. Because the magnetic field may reach 30% of its peak level throughout a region with a diameter of 4 cm[2], spheres of 4 cm diameter were created around the sample stimulation location to provide a conservative estimate of the spatial specificity achieved by TMS. These spheres were then overlaid upon each other (Figure 1). All stimulation sites were within 1 cm of target location, as described in previous publications[3,4].

Electromyography (EMG)

Electromyography (EMG) was recorded from the participant's right hand in a tendon-belly montage, to record from the First Dorsal Interosseus muscle. After scrubbing the three electrode sites with alcohol wipes, 25-mm electrodes (Kendall Neonatal ECG Electrodes Puppydog) were applied, with the ground electrode placed on the hand's carpus. In order, the EMG signal output was

processed through a D440 amplifier (Digitimer), a Humbug Noise Eliminator, 50 Hz (Digitimer) to notch-filter the data, and a CED micro1401 Mk.II A/D converter (Cambridge Electronic Design) to digitise the signal and relay it to a PC running Spike2 (Cambridge Electronic Design). Sampling rate was 5000 Hz, and bandpass filters were set between 10 and 1000 Hz.

Motor hotspot and parameter determination

The M1 coil intensity was increased and slowly moved around over the left side of the scalp until an M1 motor hotspot could be identified. Several criteria were applied to confirm the correct coil location had been reached: reliability of the MEPs, smoothness of the MEP shape, selectivity of finger movement during MEPs, and degradation of MEP response as the coil moved away from the identified spot. A subset of tested participants failed to meet all of our motor hotspot criteria, and thus did not complete the protocol and were excluded from any TMS-related analysis. The location of the motor hotspot was recorded through neuronavigation during the first session. After the first session, the recorded location was used to quickly re-confirm the motor hotspot; this also ensured the same motor hotspot was stimulated across sessions in multi-day protocols.

After determining the location of the motor hotspot, three parameters were determined for each participant: the intensities for 1mV, rMT and aMT. 1mV was determined as the intensity giving reliable and stable 1mV MEPs at rest over approximately 10 pulses; stability of the MEPs, rather than a precise mean value of 1mV across 10 pulses, was used as the key parameter in determining 1mV intensity. rMT was determined as the intensity at which 5 out of 10 pulses gave no MEP response greater than 0.05 mV.

After 1mV and rMT determination, a brief, standardised protocol was used to determine aMT[5]. A separate screen was moved towards the participant so that they could observe their own FDI EMG trace in real time from the chinrest. After the participants verbally confirmed they could see the screen, they were asked to squeeze the thumb against the index finger as hard as they could twice

to determine their maximum contraction. A sliding line on the screen was then set to 20% of maximum muscle contraction, and participants were asked to try and keep their contraction levels around that line. If this level of contraction caused fatigue or was too close to the noise level, then maximum contraction was calculated once again. This FDI-contraction set-up allowed measurement of the aMT, which we defined in this experiment as the intensity at which 5 out of 10 TMS pulses produced an MEP that was time-locked to the TMS pulse, and was followed by a cortical silent period. Presence of time-locked MEPs, rather than presence of cortical silent period, was used as the key parameter in determining aMT.

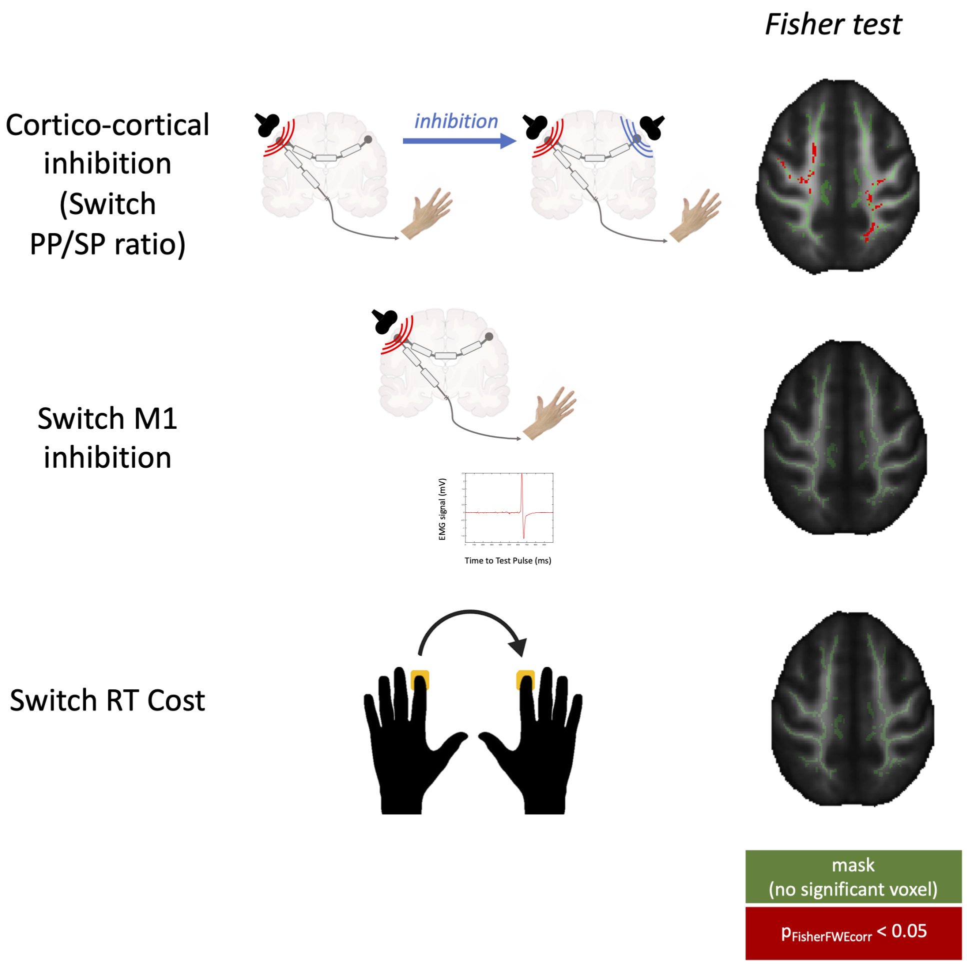

Supplementary Figure 1: **Relationships between white matter myelination, and physiological and behavioural measures.** We use joint inference and find that ppTMS-based measures of cortico-cortical interactions (i.e. switch PP/SP ratio) significantly correlate with myelin markers (peak $p_{FisherFWE} = 0.016$), whereas behavioural switch RT cost and switch M1 inhibition do not significantly correlate with myelin markers (peak $p_{FisherFWE} = 0.058$ and 0.192, respectively).

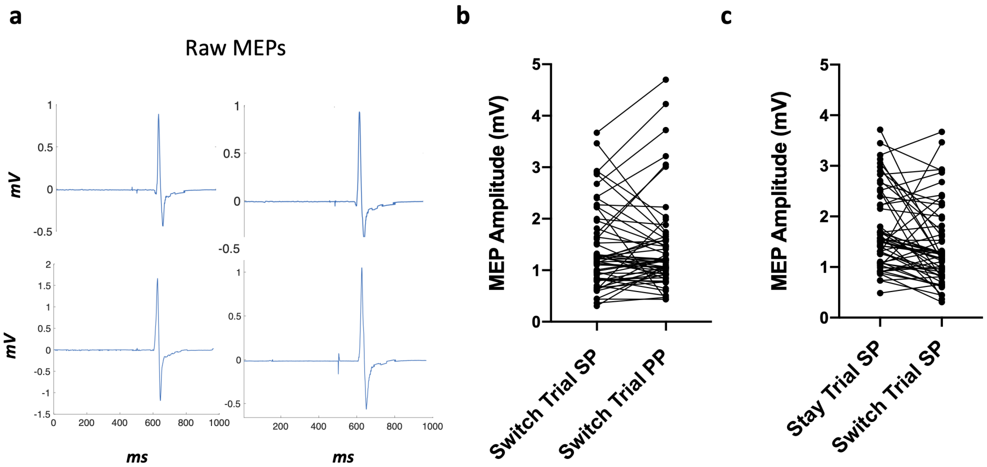

Supplementary Figure 2: **Raw Motor-Evoked Potentials (MEPs) across conditions.** A. Raw EMG traces for randomly selected MEPs from the dataset. B. Raw MEP amplitudes used to derive the switch PP/SP ratio; 29 out of 56 subjects had smaller MEPs in PP compared to SP trials during switch trials. Each data point represents a single participant. C. Raw MEP amplitudes used to derive switch M1 inhibiton. Each data point represents a single participant.

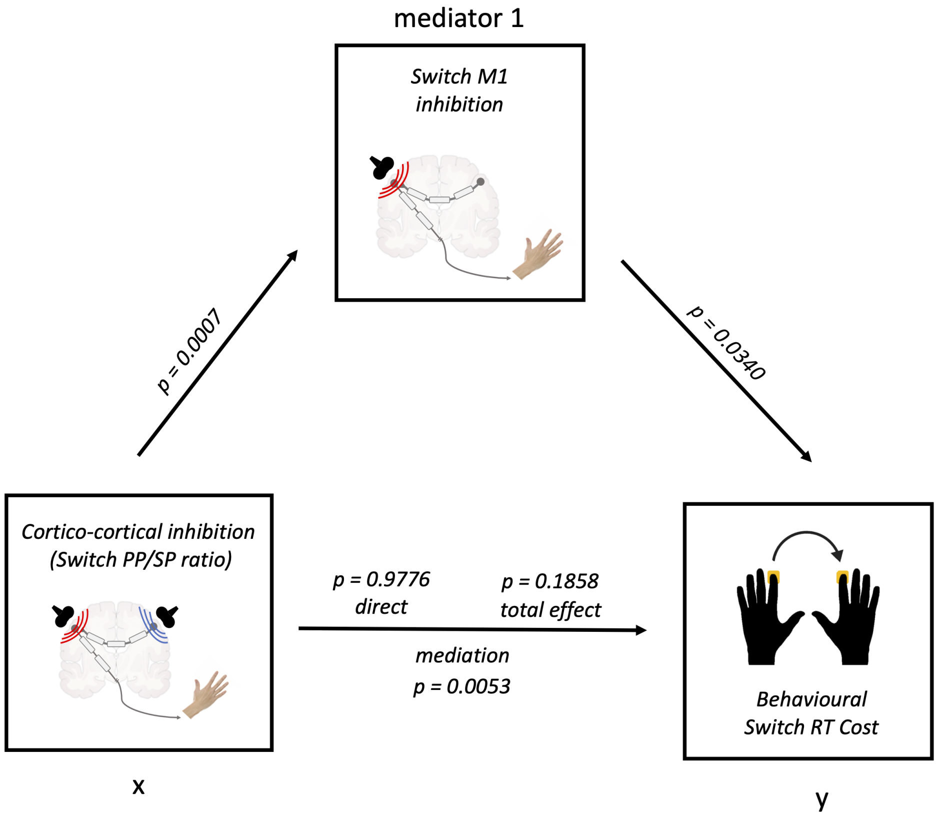

Supplementary Figure 3: **1-mediator mediation analysis.** To complement the full 2-mediator analysis, we show that 1-mediator analysis (with no neuroimaging data) yields similar results. In this analysis, we use switch M1 inhibition as a mediator of the relationship between cortico-cortical interactions (ppTMS metric) and behaviour output (switch RT cost).

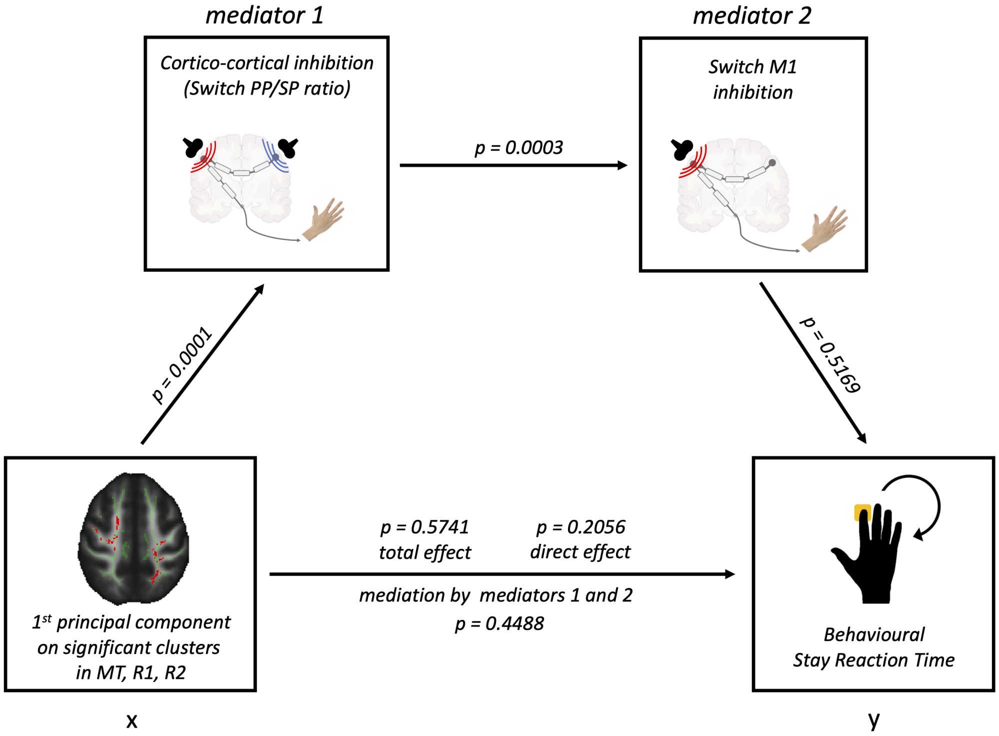

Supplementary Figure 4: **Mediation analysis for stay trial Reaction Times.** To test the behavioural specificity of the mediation analysis result, we repeated the analysis with stay trial Reaction Times as our outcome measure, and found no significant mediation effect (p=0.4488).

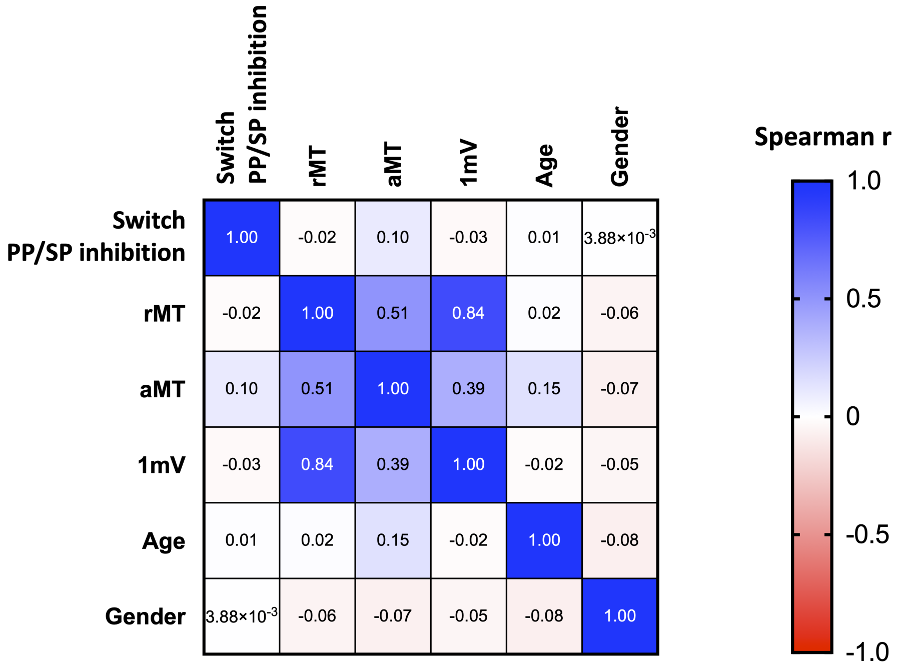

Supplementary Figure 5: **Correlations between cortico-cortical inhibition (switch PP/SP ratio), demographic factors and features of M1 physiology.** We report Spearman r correlation values for correlations of all possible pairs between: switch PP/SP ratio, resting Motor Threshold (rMT), active Motor Threshold (aMT), 1mV, age and gender. We find no significant correlation for the switch PP/SP ratio metric across any tests.

Supplementary References

1. Franz-Xaver Neubert, Rogier B Mars, Ethan R Buch, Etienne Olivier, and Matthew FS Rushworth. Cortical and subcortical interactions during action reprogramming and their related white matter pathways. *Proceedings of the National Academy of Sciences*, 107(30):13240–13245, 2010.

2. Hartwig R Siebner, Gesa Hartwigsen, Tanja Kassuba, and John C Rothwell. How does transcranial magnetic stimulation modify neuronal activity in the brain? implications for studies of cognition. *Cortex*, 45(9):1035–1042, 2009.

3. Ethan R Buch, Vanessa M Johnen, Natalie Nelissen, Jacinta O'Shea, and Matthew FS Rushworth. Noninvasive associative plasticity induction in a corticocortical pathway of the human brain. *Journal of Neuroscience*, 31(48): 17669–17679, 2011.

4. Vanessa M Johnen, Franz-Xaver Neubert, Ethan R Buch, Lennart Verhagen, Jill X O'Reilly, Rogier B Mars, and Matthew FS Rushworth. Causal manipulation of functional connectivity in a specific neural pathway during behaviour and at rest. *Elife*, 4:e04585, 2015.

5. CJ Stagg, S Bestmann, AO Constantinescu, L Moreno Moreno, C Allman, R Mekle, M Woolrich, J Near, H Johansen-Berg, and JC Rothwell. Relationship between physiological measures of excitability and levels of glutamate and gaba in the human motor cortex. *The Journal of physiology*, 589(23):5845–5855, 2011.
