# Supplementary material for: A macroscopic link between interhemispheric tract myelination and cortico-cortical interactions during action reprogramming
Source: Nat Commun. 2022 Jul 22;13:4253. doi: 10.1038/s41467-022-31687-5 (PMC9307658; doi:10.1038/s41467-022-31687-5)
Supplement: Supplementary file 3 — Description of Additional Supplementary Files [file 41467_2022_31687_MOESM3_ESM.pdf]

Description of Additional Supplementary Files

Supplementary Movie 1. Tractography of stimulated white matter fibers in 3D. Group-level map of white matter fibers stimulated in our paradigm, as estimated through DWI-based tractography.
