# Supplementary material for: A macroscopic link between interhemispheric tract myelination and cortico-cortical interactions during action reprogramming
Source: Nat Commun. 2022 Jul 22;13:4253. doi: 10.1038/s41467-022-31687-5 (PMC9307658; doi:10.1038/s41467-022-31687-5)
Supplement: Supplementary file 5 — Reporting Summary [file 41467_2022_31687_MOESM5_ESM.pdf]

nature research

Corresponding author(s): Alberto Lazari

Last updated by author(s): Mar 26, 2021

Reporting Summary

Nature Research wishes to improve the reproducibility of the work that we publish. This form provides structure for consistency and transparency in reporting. For further information on Nature Research policies, see our Editorial Policies and the Editorial Policy Checklist.

Statistics

For all statistical analyses, confirm that the following items are present in the figure legend, table legend, main text, or Methods section.

n/a	Confirmed	
☐	☒	The exact sample size (n) for each experimental group/condition, given as a discrete number and unit of measurement
☐	☒	A statement on whether measurements were taken from distinct samples or whether the same sample was measured repeatedly
☐	☒	The statistical test(s) used AND whether they are one- or two-sided<br>*Only common tests should be described solely by name; describe more complex techniques in the Methods section.*
☐	☒	A description of all covariates tested
☐	☒	A description of any assumptions or corrections, such as tests of normality and adjustment for multiple comparisons
☐	☒	A full description of the statistical parameters including central tendency (e.g. means) or other basic estimates (e.g. regression coefficient) AND variation (e.g. standard deviation) or associated estimates of uncertainty (e.g. confidence intervals)
☐	☒	For null hypothesis testing, the test statistic (e.g. F, t, r) with confidence intervals, effect sizes, degrees of freedom and P value noted<br>*Give P values as exact values whenever suitable.*
☒	☐	For Bayesian analysis, information on the choice of priors and Markov chain Monte Carlo settings
☒	☐	For hierarchical and complex designs, identification of the appropriate level for tests and full reporting of outcomes
☐	☒	Estimates of effect sizes (e.g. Cohen's d, Pearson's r), indicating how they were calculated

Our web collection on statistics for biologists contains articles on many of the points above.

Software and code

Policy information about availability of computer code

Data collection	Behavioural stimuli and Non-Invasive Brain Stimulation were delivered through MATLAB. A detailed description of the behavioural and stimulation paradigms in available in the Methods and Supplementary Methods. Full code used for data collection is available here: https://github.com/lazaral/paTMS task
Data analysis	We analyzed data using FMRIB Software Library (FSL) v6.0 and Matlab 2018b. For mediation analyses, we used the PROCESS toolbox (v3.4.1; implemented in SPSS, version 26) and BRAVO toolbox (version 2.0; MATLAB_R2018b). For preprocessing of MPM data, we used the hMRI toolbox (Tabelow et al., 2019). The R package psych (version 2.1.9; R version 4.0.0). Brainsight (version 2.3.12) and Picoscope 6 (version 2.3.12) were also used for the purposes of the study.

For manuscripts utilizing custom algorithms or software that are central to the research but not yet described in published literature, software must be made available to editors and reviewers. We strongly encourage code deposition in a community repository (e.g. GitHub). See the Nature Research guidelines for submitting code & software for further information.

Data

Policy information about availability of data

All manuscripts must include a data availability statement. This statement should provide the following information, where applicable:

- Accession codes, unique identifiers, or web links for publicly available datasets
- A list of figures that have associated raw data
- A description of any restrictions on data availability

Data used in this study is only available upon request due to data protection considerations.

Field-specific reporting

Please select the one below that is the best fit for your research. If you are not sure, read the appropriate sections before making your selection.

☒ Life sciences ☐ Behavioural & social sciences ☐ Ecological, evolutionary & environmental sciences

For a reference copy of the document with all sections, see nature.com/documents/nr-reporting-summary-flat.pdf

Life sciences study design

All studies must disclose on these points even when the disclosure is negative.

Sample size	No statistical method was used to predetermine sample sizes, as it is particularly difficult to carry out power analyses that account for cluster-wise inference. This study also employs multi-modal statistical tests, which further complicates matters as any power analysis would need to account for dependence across imaging modalities. Therefore, we based our sample size on recommendations from previous literature (De Santis et al., 2014) and on previous studies finding similar effects (e.g. Neubert et al., 2010).
Data exclusions	One participant was excluded as the MPM scan was heavily corrupted due to movement artifacts; one participant was excluded due to lower quality signal in the MPM scan, which resulted in poor registrations with other modalities.
Replication	We do not attempt any direct replication within this manuscript. Instead, to maximize our statistical power, we opted to pool all subjects into a single analysis, resulting in a much larger sample size (n=64) than any previous study using similar TMS paradigms (e.g. n=16 in Neubert et al., 2010).
Randomization	We used a within-subject design and no randomization was performed. Randomization and blinding are not relevant to this study, as subjects were not allocated to different groups.
Blinding	We used a within-subject design and no randomization was performed. Randomization and blinding are not relevant to this study, as subjects were not allocated to different groups.

Reporting for specific materials, systems and methods

We require information from authors about some types of materials, experimental systems and methods used in many studies. Here, indicate whether each material, system or method listed is relevant to your study. If you are not sure if a list item applies to your research, read the appropriate section before selecting a response.

Materials & experimental systems

n/a	Involved in the study	
☒	☐	Antibodies
☒	☐	Eukaryotic cell lines
☒	☐	Palaeontology and archaeology
☒	☐	Animals and other organisms
☐	☒	Human research participants
☒	☐	Clinical data
☒	☐	Dual use research of concern

Methods

n/a	Involved in the study	
☒	☐	ChIP-seq
☒	☐	Flow cytometry
☐	☒	MRI-based neuroimaging

Human research participants

Policy information about studies involving human research participants

Population characteristics	64 healthy participants (36 female, 28 male; mean age across male and female participants: 24.69 years) participated in the study. All participants were self-assessed right-handed and their handedness was further confirmed through the Edinburgh Handedness Inventory (mean EHI score: 88.65).
Recruitment	We recruited through advertisement of the study to the general public in the local community (in Oxford, UK). All advertisement materials were approved by the Central University Research Ethics Committee (CUREC) priori to circulation. All participants were screened for TMS and MRI safety, received monetary compensation for their participation, and gave their informed consent to participate in this study.
Ethics oversight	All study procedures were reviewed and approved by the local ethics committee at the University of Oxford (Central University Research Ethics Committee (CUREC)), and followed the Declaration of Helsinki.

Note that full information on the approval of the study protocol must also be provided in the manuscript.

Magnetic resonance imaging

Experimental design

Design type	Multimodal microstructural imaging at rest; within-subject cross-sectional design.
Design specifications	N/A (no behaviour during the MRI scan)
Behavioral performance measures	N/A (no behaviour during the MRI scan)

Acquisition

Imaging type(s)

Structural (T1-weighted structural imaging), Diffusion (Diffusion-Weighted Imaging) and other microstructural modalities (Multi-Parameter Mapping).

Field strength

3T

Sequence & imaging parameters

The T1w sequence (TR = 1900 ms, TE = 3.96 ms, voxel size = 1mm isotropic, GRAPPA = 2) had a large Field of View (FOV = 256mm^3) to allow for the nose and intertragic notches of the ears to be included in the image to facilitate later neuronavigation of the TMS coil to the target position.

Diffusion-weighted Echo-planar imaging (EPI) scans (TR = 3070 ms, TE = 85.00 ms, FOV = 256mm^3), voxel size = 1.5mm isotropic, multiband factor of 4) were collected for two b-values (500 and 2000 s/mm^2), over 281 directions. An additional 23 volumes were acquired at b=0, 15 in anterior-posterior (AP) phase-encoding direction and 8 in the posterior-anterior (PA) phase-encoding direction.

The Multi-Parameter Mapping (MPM) protocol (Weiskopf et al. 2013) included three multi-echo 3D FLASH (fast low-angle shot) scans with varying acquisition parameters, one RF transmit field map (B1+map) and one static magnetic (B0) field map scan, for a total acquisition time of roughly 22 minutes. To correct for inter-scan motion, position-specific receive coil sensitivity field maps, matched in FOV to the MPM scans, were calculated and corrected for (Papp et al. 2016). The three types of FLASH scans were designed to be predominantly T1-, PD-, or MT-weighted by changing the flip angle and the presence of a pre-pulse: 8 echoes were predominantly Proton Density-weighted (TR = 25ms; flip angle = 6 degrees; TE = 2.3-18.4ms), 8 echoes were predominantly T1-weighted (TR = 25ms; flip angle = 21 degrees; TE = 2.3-18.4ms) and 6 echoes were predominantly Magnetisation Transfer-weighted (MTw, TR = 25ms; flip angle = 21 degrees; TE = 2.3-13.8ms). For MTw scans, excitation was preceded by off-resonance Gaussian MT pulse of 4 ms duration, nominal flip angle, 2 kHz frequency offset from water resonance. All FLASH scans had 1 mm isotropic resolution and field of view (FOV) of 256x224x176 mm^3. The B1 map was acquired through an EPI-based sequence featuring spin and stimulated echoes (SE and STE) with 11 nominal flip angles, FOV of 192x192x256 mm^3 and TR of 500 ms. The TE was 37.06 ms, and the mixing time was 33.8 ms. The B0 map was acquired to correct the B1+ map for distortions due to off-resonance effects. The B0 map sequence had a TR of 1020.0 ms, first TE of 10 ms, second TE of 12.46 ms, field of view (FOV) of 192x192x256 mm^3 and read-out bandwidth of 260 Hz/pixel.

Area of acquisition

Whole-brain

Diffusion MRI ☒ Used ☐ Not used

Parameters

Multi-shell diffusion-weighted imaging was used. Two b-values (500 and 2000 s/mm^2) were corrected over 281 directions. An additional 23 volumes were acquired at b=0, 15 in anterior-posterior (AP) phase-encoding direction and 8 in the posterior-anterior (PA) phase-encoding direction.

Preprocessing

Preprocessing software

MRI scan pre-processing, analysis and statistical comparisons were performed using FMRIB Software Library (FSL, v6.0), except for the MPM quantitative map estimation step which was carried out using the hMRI toolbox implemented in Matlab-based SPM12, as described in (Tabelow et al. 2019).

Normalization

To bring all volumes into a common space, native FA volumes were skeletonised with Tract-Based Spatial Statistics (TBSS), and the skeletonisation transforms were subsequently applied to MPM-to-DWI registered volumes. Group-level analyses were then conducted in skeleton space for all data.

Normalization template

We used a TBSS-derived skeleton based on our subjects as our common space for group-level analyses.

Noise and artifact removal

The topup tool was run on average images of AP b0 volumes and PA b0 volumes. The resulting susceptibility-induced off-resonance field was used as an input for the eddy tool (Andersson and Sotiropoulos 2016), which was run with options optimised for multiband diffusion data to correct for eddy currents and subject movement.

Volume censoring

N/A (structural data)

Statistical modeling & inference

Model type and settings

Voxelwise joint inference was performed through Permutation Analysis of Linear Models, which implements a voxelwise

Fisher test with the following equation as described in Winkler et al. 2016.

Effect(s) tested	Correlation with PP/SP ratio was the main effect of interest; control analyses were run using additional behavioural and physiological regressors as described in the Methods and Supplementary Methods

Specify type of analysis: ☒ Whole brain ☐ ROI-based ☐ Both

Statistic type for inference (See Eklund et al. 2016)	Cluster inference was performed with a cluster-forming threshold of $t>1.67$ (equivalent to $p<0.05$, based on the degrees of freedom) in all instances.
Correction	Family-wise error cluster correction (with a cluster-forming threshold of $t>1.67$, at the 5% familywise error level).

Models & analysis

n/a	Involved in the study
☒	☐ Functional and/or effective connectivity
☒	☐ Graph analysis
☒	☐ Multivariate modeling or predictive analysis
